# Can music serve as a “cultural immunogen”? An explorative study

**DOI:** 10.3402/qhw.v8i0.20597

**Published:** 2013-08-07

**Authors:** Even Ruud

**Affiliations:** Department of Musicology, University of Oslo, Oslo, Norway

**Keywords:** Music, health, narratives, culture, music therapy, health musicking

## Abstract

The aim of this study is to explore how people in contemporary society may apply music in their everyday life to improve their health and well-being. Through a series of qualitative interviews, informants gave their narratives about how music had become a part of their health practice. Six narratives concerning this type of everyday musical self-care are presented, and the following questions are sought to be answered: What kinds of musical practices do people apply in order to regulate their health and promote their sense of well-being? What kind of generative health mechanism can we observe or theorize when people use music to enhance their well-being? What kinds of rituals, contextual circumstances and personal health beliefs are operating in these situations? The findings suggests that some people may sing, participate in a choir, dance to music, compose songs, play precomposed music, or play in a band as part of a reflexive strategy to improve their health and well-being. Further analysis also identified six generative factors that may contribute to the immunogen functions of music: A pragmatic concept of music, music as a social and emotional resource, music as a supportive self object, musical competency, rituals, and locus of control. These findings may have implication for the field of music therapy as it will fill the gap between the clinical use of music done by professional music therapists and the everyday “musicking” performed by people outside the institutional practice.

The current use of music in medical care, rehabilitation and special education has its roots in a long historical tradition going back to the ancient Greeks (Horden, 2000). In the music theory of Pythagoras (and later Plato), music was believed to serve curative functions and represent a means of maintaining psychological balance in everyday life. It is suggested that Pythagoras himself (West, 2000), as a religious leader, practiced a sort of “musicking” in the evening in order to cleanse his mind from everyday noise and purify his thoughts, to restore his balance or harmony before going to sleep. Additionally, he used music in the morning to prepare himself for the coming day (Schumacher, 1958). In other words, Pythagoras seems to have practiced a very familiar functional use of music: musicking^1^ as a technology to regulate a body–mind relationship.

As Ansdell (in press) observes, this dual tradition of applying music both as a medical cure and as a more preventative or health-promotive practice has persisted throughout Western history, although both aspects of music seem to have faded during the 19th century due to the rise of positivism (Kümmel, 1977) and, later, the dominant bio-medical model within health care (see also Ruud, 1990).

With its establishment over the decades since the mid-20th century, modern music therapy has come to encompass most of the fields of practice already familiar from the health services, community music, and special education (Wigram, Bonde, & Pedersen, 2002). Currently, there seems to me to be two main trends, going in different directions. The medical model attracts music therapists towards an evidence-based practice, whereas a community-based approach operating within an ecological and systemic ideology invites music therapists to work outside established health institutions (Ansdell, in press; Pavlicevic & Ansdell, 2004; Stige & Aarø, 2012). Each of these trends is seen within music therapy practices to be utterly dependent upon the musical skills, theoretical knowledge, and communicative abilities of the music *therapist*.

However, music *therapy*, seen as a *discipline* dedicated to the relationships among music, health, and the individual, is broader still and engages with the whole range of musical behaviour devoted to enhanced well-being. Thus, the everyday practices of people using music to enhance their health and well-being independently of a music therapist—that is, as a form of self-help technology—is also very much of interest. In this article, I will present six narratives concerning this type of everyday musical self-care in relation to the following questions: What kinds of musical practices do people apply in order to regulate their health and promote their sense of well-being? What kind of generative health mechanism can we observe or theorize when people use music to enhance their well-being? What kinds of rituals, contextual circumstances, and personal health beliefs are operating in these situations?

## A cultural immunology?

Within the field of health psychology, the study of health-related behaviours tends to focus upon behaviour that may protect health, or so-called behavioural immunogens (Matarazzo, 1984, in Ogden, 2000, p. 13; Matarazzo & Leckliter, 1988). I have suggested elsewhere, along these lines, that we conceptualize music as a form of *cultural immunogen*, along with other behavioural immunogens, such as dental hygiene, the use of safety belts, good sleeping habits, and so on (Ruud, 2002). One of the reasons why I coined this term was because of the lack of concern for the arts and culture in the field of health psychology. A “cultural immunogen” thus implies the handling of cultural artefacts or artistic expressions within the context of health-related behaviour.

Within the field of medicine or immunology, an immunogen is a specific type of antigen, or a substance that is able to provoke an adaptive immune response if injected on its own. In health psychology, then, a “behavioural immunogen” must be understood in a metaphorical sense, as a sort of protective behaviour, as opposed to a behavioural pathogen—that is, a harmful behaviour that is damaging your health (smoking, excessive drinking, driving without a safety belt, and so on).

Health psychologists supply a lot of evidence regarding how our attitudes and behaviour towards our own health practices will influence our state of well-being, in relation to topics, such as eating, exercising, addiction, and so on. There seems to be little interest in the areas of behaviour related to cultural consumption—that is, the interaction between the arts and health-related topics, such as stress, pain, and anxiety. However, there is a vast amount of research and literature in the area of music therapy itself (see Wigram, Bonde, & Pedersen, 2002, for an overview).

Another theoretical inspiration for this article is the work of Antonovsky (1987) on salotugenetic factors influencing our health. In his research, Antonovsky outlined how a sense of coherence in life made a crucial difference to health. One aspect of this general sense was how meaningful life was felt to be; other aspects included life's predictability and our ability to handle it. All of these aspects are relevant when it comes to how music, or cultural production and consumption in general, can influence our health and even our longevity (Bygren et al., 2009).

## Six narratives on music as self-care

Relatively few studies have focused upon the use of music in everyday life as a cultural immunogen, that is, outside the professional use of music in music therapy, (but see, among others, Batt-Rawden, 2007; Batt-Rawden, DeNora & Ruud, 2005; Bonde, Ruud, Skånland, & Trondalen, 2013; Bossius & Lilliestam, 2012; Ruud, 2002; 2009; Skånland, 2012). This is likely because, as mentioned, music therapy as a profession has traditionally (and unsurprisingly) stressed the importance of a professionally trained music therapist to the overall music therapeutic process. Lately, however, the emerging field of music and health (see MacDonald, Kreutz & Mitchell, 2012), in tandem with recent trends in resource-oriented and community music therapy, has revealed new possibilities for widening the compass of music therapeutic interventions to include the intersections between clinical work and people's other engagements in society.

There is a vast literature on music as therapy where the music is administered within a contract-based relationship between a music therapist and a client. A simple search on the internet will, for instance, also reveal extensive literature on the physiological and neuropsychological effects of music. There are reports in the media as well as speculative literature within the New Age paradigm that point to music as a source of general well-being or a path to esoteric knowledge. And there are, lastly, ethnographic accounts from other cultures of how musical practice is considered to have implications for health and ill health (Gouk, 2000). In what follows, I will present six narratives on everyday health musicking that I have gathered during my years conducting interviews for research purposes, in the interests of turning focus to the health-related potential of the everyday use of music (or musicking).

Informants were sought for through the internet, or they approached the researcher after hearing about the project. Statens Datatilsyn approved the project and the informants gave their informed consent to participate in the study. No specific selection criteria were chosen, and the informants did not represent a certain population, a specific health problem, or had any defined musical background. The narratives are based upon qualitative interviews, as defined by Kvale (1997), i.e., the aim was to explore the informants’ life world or reflections around their use of music to meet a specific health issue. The interviews were tape-recorded and transcribed. No standard questionnaire was used. The six narratives presented here were selected in order to cover different aspects of musicking, ranging from choir participation, playing an instrument, listening to music, singing, a song–dance ritual, and playing in a band. In alignment with the explorative nature of this study, the purpose of this selection of narratives was to demonstrate potential health generative effects of a variety of health musicking practices. The analysis of the data was based upon an open coding approach (Strauss & Corbin, 1995) seeking to identify themes and categories relevant to explain the nature of health musicking. Further axial coding (ibid.) led to the construction of six “generative mechanisms” as presented in the discussion: a pragmatic concept of music, music as a social and emotional resource, music as a supportive self object, musical competency, rituals, and locus of control.

### The choir as social capital

The first narrative involves a woman who built up a social network through the establishment of a local choir in her neighbourhood. Though this professional health care worker had been successfully treated for breast cancer, she found herself in an insecure life situation afterward and wanted to respond to her diagnosis by doing something meaningful with her life. She started taking piano lessons but found it too difficult to combine practicing with childcare at home. She then resumed singing lessons (she had taken them earlier in her life) and enjoyed them but wanted something more—something she could share with others and possibly use as a way to support her children in case her illness should develop in the wrong direction. In the latter regard, she wanted more contact with other parents in her children's school, and she came up with the idea of starting a choir for them. She began by contacting a friend with some musical background, and eventually 25 women joined the choir. They then had the good fortune to find a conductor who seemed to be an ideal fit.

When I asked her about what she herself had gained from these efforts, she underscored first of all the pleasure she felt regarding how she and her friend managed to go through with this initiative, the joy of singing in the choir, and the musical knowledge she has derived from it. She also emphasized the social part as something she would never have known “and which has ripples. Exactly this [benefit] about building a network is something at least I have managed through this [choir]”. This network brought her new friends with whom she could discuss personal matters—friends who were supportive and gave her a feeling of mastery over her life. These relationships also grew to encompass her family and children.

When I asked her if the choir had any more direct impact upon her handling of her own illness or even simply kept her thoughts on other things, she said:

It is one thing that it takes away the thoughts. Another aspect, which in a way is always there … because I am quite goal-directed … actually, I think, like if I should die, if my illness comes back and I am gone, I have in any case built a network. This is a potential network around my children and husband, who is not as social as I am.

The choir also became so accomplished musically that they were invited to perform at school gatherings and in the local church. This gave choir participants social leverage and a position of visibility with regard to the school administration, which in turn made it possible for them to negotiate access to necessary educational resources for some of the children or to impact the educational content in the school.

### Playing Chopin to explore feelings of loss and grief

A woman told me how she felt while playing a Chopin nocturne while her grandmother was terminally ill in the hospital:

While I sat there playing [Chopin's Nocturne in E minor, Opus 72, No. 1], I suddenly experienced how the music expressed what I felt. I did not sit down in order to play or express those feelings. Rather, it was like the feelings came to me from the music … both the evident sad feelings … because the piece carries this heavy expressiveness …. But, in addition, there are some parts in the piece which are a bit lighter, brighter, in a way, happier. And then I realised that I could feel a bit of that also, because I could look back at what had been good. She really had had a good life …. The musical piece also has parts I felt like [were] angry, in a way—they became, like, ‘This is not fair, I am angry’. But also her fight against the illness, because I knew how she was so angry, it felt so wrong. It was difficult to accept this. So while I was playing, I felt many things, and I felt, ‘This is what I feel now’. And then I could let go of my feelings through playing this piece.

Here, we are able to watch this woman locate feelings in the music that match her own, and the music becomes a way to both explore and express those feelings. She indicated that her perspective towards the music changed as she played it, and that she consciously tried to discover what she might find in the different parts of the music. At the same time, she tried to relate to and interpret the music in the context of her grief.

She went on to say that she not only found grief, anger, and hope but also gratitude for what had been, and that some parts of the music became so concrete for her that she was able to hear teardrops falling. On the other hand, the gentler, lighter passages returned to her all the good she had experienced and helped her to integrate her mixed feelings and move on in her process of grieving.

In the end, she recognized that another aspect of her grieving was cognitive, and that this went hand-in-hand with the emotional aspect. While music helped her to gain access to her feelings, it was not sufficient. She also had to treat the process intellectually, and she had to talk to someone to do so.

### Listening rituals for overcoming grief and life crises

A woman contacted me to tell her story about how she had used music listening to overcome a significant life crisis after having lost her husband. After he passed away, she experienced episodes of panic anxiety, and she watched a conglomeration of emotions, such as sadness, anger, guilt, and shame lead her into her crisis. She admitted that she probably should have sought professional help, but she decided instead to use music to handle her emotions, and she began to divide her large collection of CDs into categories of emotion. When she described this attempt to relate particular songs or artists to a single emotion, she resorted to a metaphor and conceptualized her collection as a chest full of emotion drawers. When she pulled out a drawer and played a CD, she tapped into the related emotion. She explained how painful this process was but noted that she did not use music to avoid what was unpleasant but instead to confront it. She found it, then, bittersweet to explore and indulge in her painful feelings through music, but from this ordeal emerged a sort of acceptance—the music received her feelings without being judgmental, almost like a friend (or a therapist):

It is easier to be in such a situation when there is someone close to you to stand by you. But if you don't have anybody, or if you perhaps do not want to bother other people …. As a grownup, you often have to stand alone. I believe so. And this is also an acknowledgment. In the end, we all stand alone …. Then, music has been a helper.

This woman had a large collection of CDs, and her musicking engaged with a great variety of artists and genres—rock and popular music, film music, classical music and ethnic music. She used another metaphor—her “musical home pharmacy”—for her music collection, to which she turned every day after work for a number of years. She reported developing a special “musical vigilance” in this period, as she found meaning in lyrics and music that she turned to for self-help. Even when she listened to the radio, she wore these “emotional antennas”.

### Singing, physical well-being and anxiety reduction

An 85-year-old man contacted me after being told about the project. He had been the victim of a serious lung disease as a small child, and it had developed into asthma. When his family, during the 1920s, moved to a colder climate in Norway, he often suffered asthma attacks. Nevertheless, he was deeply involved in singing from early on. Inspired by his father, who used to entertain him with his pleasant baritone voice while my informant was in bed with bronchitis, he discovered that his mood changed for the better when he sang to himself as well. He could not sing, of course, while in a state of acute asthma, which restricted his ability to breathe in, but he sought to improve his lung capacity by systematically practicing his singing and working on his breathing. “Singing—and it was quite a lot—became a major part of my everyday life. I learned to sing and to do breathing exercises and how to use my lungs all the way down”, he said while demonstrating how his breath could move the lower part of his stomach. “I can still be pretty flexible there, so I think this had a great influence on my health”, he added, noting that his asthma disappeared when he turned 18. Many years later, when he had his lungs X-rayed, the medical examiner could still see traces of his lung disease but could also confirm that not many people would be likely to keep up with him on a walk through the forest.

His clear lungs also indicated the work that he had done on his voice as a student, and he continued to pursue this interest in his adult life. As he discussed his vocal ability and conflicted experiences with perfect pitch (which sometimes led him astray), he suddenly exclaimed: “It was enourmously joyful, life-confirming, encouraging … I don't know how to express it. But my singing had a lot to do with my quality of life”.

This turn in the conversation led him to reflect upon how music is able to change our mood, to calm us, to brighten things up, to comfort us, and give us hope in life. Unsurprisingly, the old theologian had quite a repertoire of hymns that he could utilize to regulate his mood. He had suffered from several serious diseases since his childhood and even had a heart attack, and his awareness of the constant possibility of sudden death created a need in him for music to be a supportive and nurturing presence. When I asked him if his singing did something to his thinking—if he felt that music changed anything—he agreed that it did. He then told me that, during the war, he had been visited by the Gestapo under suspicion of keeping weapons in his house, a “deadly serious and breathtaking” experience that visited him for many years while he slept. After these nightmares, he was too disturbed to be able to sing. But in general, he assured me, “I am able to sing while in bed. I do that from time to time, with great pleasure”. I asked him if he was consciously trying to change his mood by doing so, and he responded, “Yes, definitely. But I have to wait for a while. After a nightmare, I have a terrible palpitation of the heart, and my pulse is irregular. Sometimes even my ears are beating.” When his body had returned to its normal state, he would be able to sing: “Yes, I use the singing. I lie in the bed and sing. Usually I keep my hymn book in the bed beside me. I nearly always keep the hymn book beside me”.

In this case, music was clearly used as a means of literally expanding and strengthening lung capacity as well as regulating mood in order to prevent anxiety or restore a state of normal physiological functioning after a nightmare.

### *Song*–dance rituals as a catalyst of stress and anger

A woman told me that she took up a song–dance ritual after she could no longer run as exercise, which she had done as a way to regulate her level of stress. I asked her how she actually felt when she was stressed and whether she could describe her bodily state before and after singing. “You seem to be pretty aware of this?”, I asked her.

Yes. Music gives you a sort of release. You are in a state of everyday stress because of your work or because you demand something of yourself … First, I have to listen to some fast music and then some more quiet music. I sing and dance at home alone by myself to the fast music. I prefer to use the headset in order not to disturb the neighbours too much. And then, it seems, the adrenaline comes and gives you a kick. And you get a sort of release and then a sense in the body when the stress has somewhat left the body and you can put on some more quiet music. It actually happens [that] I sing so loud that I can feel it in my vocal chords afterwards, that ‘now I have really been shouting’.

I asked her if she could tell me more about this self-absorbed feeling—that is, “where” she was when she was in this state. She responded, “I enter the music totally. I forget everything. And it is a very good feeling. Even though the headphones may harm my ears”. I asked her if she feels the stress she is battling in her head or in her body.

It is rather the body that is somewhat stressed—for me it is usually the stomach. But I get a fairly instant bodily relaxation. I don't get a headache. I'm not that kind of person. But I have too many thoughts and it feels good to get out of the thoughts. Music takes control, and it is such a good feeling, because you tend to forget everything you have been thinking about.

I asked her to tell me more about her feelings.

I do actually listen when I am angry. Then I also need the loud music to balance my anger. Even though I have lost my anger during these last years, it used to be a problem earlier. So I do not need that kind of music as much as I used to. Because earlier, I had a real need to ‘get it out’, as a counterweight to the anger I felt within. But it has become less, though it is still there when I want to relax. Earlier it was more anger; now I do it because I am stressed and it makes me feel good.

This could be interpreted as an example of a strict procedure or musical ritual in the service of a health-performing behaviour. One notices one's state of stress and selects music to address it according to a fast–slow/loud–soft format. In this way, my informant has learned to sort out her anger as well.

### Rock band participation and songwriting against depression and social phobia

Another informant was brought to my attention by a fellow music therapist who for many years had been working with the rock band format with female inmates, both inside the prison and in the community after their release. One of these women, thanks to this project, had been inspired to start her own band.

Now in her 40s and despite encountering serious social and emotional difficulties, she was nevertheless able to realize her adolescent dreams of playing in a band, writing lyrics, putting music to words, performing in public, and recording her songs. When I called her to make an appointment, she mentioned that she saw music as something that gave her “health”. When I met with her, I asked her about this:

That's right, because of my emotional problems, it's really affecting my health, especially when you are feeling down. Sometimes it gets so bad, I have to pull down the curtains and go to bed or lay down on the sofa with the blanket over me. You don't want to see anyone, or talk to anyone or do anything. The only thing that can get me up from bed is if I can imagine the music. So to me, this is a real help psychologically. For then I can manage. Sometimes it is a real pain to get out of the door—to stand up, dress and leave the house. But when I finally have managed to get to the rehearsal, even if it has been very hard sometimes, I have managed because I have comforted myself [by] telling myself I will feel much better when I have left the house. Even my son has told me: ‘Mom, get down to the rehearsal. You will feel much better when you have been there’. At that moment I feel this [interaction] as fussy, but he has actually encouraged me to go. Even if I didn't have the energy. And I have never regretted it—I have always been happy I did go anyway. It feels like a personal victory … and I was in a much brighter mood when I got back home. And I also became more active—I did not go back to the sofa. I can do a lot of housework when I come home, just because I have been away.

Music seems to have become the only means of getting her off the couch; it gave her the courage and energy to go to the band rehearsal. This also seemed to represent a major step in overcoming her confusion and depression. With the band, performing her own songs, she managed to forget her own problems, to overcome her social phobias and enjoy her friendships with the other musicians and her relationship with her teenaged son. “If it hadn't been for music, I would have been even more depressed”, she added.

I asked her if she forgets her problems when she is rehearsing with the band.

I am not worrying about problems, I am just concentrating on the music. I concentrate on my own lyrics, getting really involved, because I am really eager to find out how to construct the tune—how to perform and things like that. You tend to get so involved in music that you forget your problems. And to forget your problems for a couple of hours—on a Monday!—I felt wonderful. And I did not worry any more when I came home at night. During the day, problems could lie there and grow, but never after the rehearsal.

Aside from the social and mood-enhancing effects of the band rehearsal, she also emphasized that the act of composing music and writing lyrics helped her to clear up her “confusion”, which she attributes to her emotional problems. I asked her about exactly what happens to her confusion when she is concentrating on her music: “Does the confusion disappear?”

Yes, when I have got things down on paper, I feel like I have lost ten pounds. I have many thoughts spinning around in my head, and it helps me on the psychological level to write these thoughts down. Because I get to write down how I really feel about things. It really helps me psychologically. Also, [it helps] to go to the rehearsal and play the music.

It appears, then, that music gives her a context within which to be both precise and honest about how she actually feels. Through music she is able to sort out what is important among her confused thoughts and feelings, develop a tolerance for them, and express them through her writing. Going to the rehearsal completes this process by enabling her to elaborate upon, express and share her thoughts and feelings with her band mates and an audience.

I also asked her if she sometimes listens to music specifically to change her mood.

Yes … it helps me a lot to listen to music. Because, mentally, if you are able to get up from the sofa and put on some music … [In the past] I have consciously chosen music with lyrics … that remind me of my situation. And then I start to cry, in order to really cry. Other times I can put on some music that puts me in a better mood—music with real force—in order to change my thoughts into something else. And to forget about a lot of problems. This because I actually manage to get involved in the music I am listening to. So I am somewhat conscious about what I actually listen to.

It seems here that music is used on a regular basis to refocus one's attention or address and align oneself with troubling memories in order to spark an emotional catharsis. This gives evidence to how music may also be used to change one's mood or forget about one's worries.

## Discussion

These six narratives describe the transformation of community music practices, such as choir singing or playing in a rock band, listening to and playing music, writing songs, dancing while listening, and singing to express oneself (and improve one's breathing)—into “health musicking practices” (Stige, 2012). There is no such thing as a “therapeutic music” in and of itself, as I discuss in what follows. How any given music affects us will depend not only upon the music but also upon our own listening histories, our musical identities, associations and memories, and the social context within which our listening takes place (see also Ruud, 2011).

Note, as well, that I will not be addressing here the possible harmful effects of music (and noise) due to excessive volume, or situations where people are disturbed by other people's music, or unwanted background music, or the ways in which music can be used to isolate oneself and/or reinforce negative thoughts and antisocial behaviour (see also Ruud, 2005).

My point of departure here was instead to present and discuss narratives about the use of music to regulate health. I am interested in both how this health musicking worked and under what circumstances it was possible. As we have seen, when music is applied to help regulate our health, we do not go to the pharmacy to buy a certain piece of music, as we might expect from a mechanistic model of biomedical thinking. Instead, we have seen in the previous examples some of the ways in which certain individual and idiosyncratic conditions or presuppositions have to be present in order to liberate music's immunogen function.

Can we, then, draw some general knowledge from these narratives in order to describe the conditions within which music can be seen as a cultural immunogen? Following the analysis of the interview, I also identified six conditions, or assumptions, about what might contribute to a health musicking process. My data are not sufficient to support definitive conclusions about such contextual factors, or “generative mechanisms”, but I hope that this discussion provides a starting point for future research in this regard.

### A pragmatic concept of music

It seems like my informants all draw upon a pragmatic concept of music—that is, they do not regard music as solely an aesthetic object, the pleasures of which must somehow transcend the everyday. To them, instead, music seems to be intertwined *in* the everyday, as something to enjoy, relax with, regulate stress with, and so on. This characteristic seems to accord with much recent research on the everyday uses of music (Bossius & Lilliestam, 2012; DeNora, 2000).

There are many ways of conceptualizing music. Different disciplines within musicology are linked through shared underlying assumptions about the very nature of music, or what Philip Bohlman calls the “metaphysical assumptions of music” (Bohlman, 1999), but variations abound nevertheless. Ontologies of music—that is, what we think music most fundamentally is or is not—are also constructed and articulated differently within different music therapy traditions. To the positivist music therapist, music is understood and described as “regular vibrations” effected by energy-producing sound waves, while therapists inspired by New Age philosophies may see music as a reflection of “cosmic vibrations”. To other music therapists, the essence of music is its link to sensuality and feelings; still others see processes of cognition at the heart of the musical experience.

In general, I will argue against any essentialist thinking about music, whereby musical meaning is mechanistically transferred from a composer to the score, and hence to the listener via its performance. Ultimately, we simply need a model of understanding music that encompasses the process of reception by the listener as an essential part of the construction of musical meaning.

Music therapists have traditionally resisted a concept of music as “work” and have instead embraced more processual conceptions of music, where contextual, music-structural and individual circumstances influence its interpretation and experience. Lately, Christopher Small's thoughts about musicking (Small, 1998), as well as the related notions of “affordance” and “appropriation” (Clarke, 2003; 2005; DeNora, 2000) have been widely embraced by music therapists as well. Small emphasized how music must be understood as a *practice* and a *process*—as something we do—rather than as an object. This has many implications for our understanding of how meaning is produced while we engage with music in an active rather than a passive fashion.

As we have seen, contextual and situational circumstances play a major role when musical meaning is negotiated. This brings us to the notion of “affordance”, which was initially part of James J. Gibson's ecological theory of perception (Gibson, 1979), which seeks to shed light upon any interaction between perceiver and environment. Any given environment affords a number of actions and perceptions, according to Gibson; musicologist Eric Clarke takes this to mean that “the affordances of an object are the uses, functions, or values of an object”—that is, the opportunities that it offers to a perceiver (Clarke, 2003, p. 117). Clarke then emphasizes how perception and action are inextricably linked and points to the dialectical relationship between an organism and its environment, observing that this interaction is “neither simply a case of organisms imposing their needs on an indifferent environment, nor a fixed environment determining strictly delimited behavioral possibilities” (ibid., p. 118). If there is always a social component affecting the range of possibilities inherent in socially embedded objects like music (Clarke, 2005, p. 38), the musical affordances offered by a specific piece of music will be appropriated by the listener within the “ecology” of the listening situation in question.

The concept of musicking recognizes that the context of performance, and one's idiosyncratic relationship to any given musical situation, is crucial to our decoding and interpretation of music and therefore influences the music's “effects”. This is in line with much contemporary music ethnography, which engages with the ways in which we make use of music to express symbolic meaning and core values (Feld, 1982) or use music to create social boundaries (Stokes, 1994) or construct identities (Ruud, 1997).

When sociologists of music started to talk to people in addition to theorizing the relation between music and society, it became obvious that music serves a whole spectrum of everyday needs. As music sociologist Tia DeNora observes in accordance with researchers from music psychology and cultural studies, music is present in a variety of social and personal contexts where mood is regulated, attention is focused or energy is canalized (DeNora, 2000). Music creates an emotional and cognitive context that is conducive to a feeling of well-being or a state of alertness or relaxation in accordance with the needs of the situation. Sociologically speaking, musicking represents a means of regulating the relations between the person and the situation, between our psychological state and the demands that stem from our surroundings. As we have seen in the above narratives, this regulatory role of music seems to be fundamental to its immunogen function. Within this frame of thinking, the use of music to regulate ones thoughts and emotions is theorized as a form of “technology of health”.

The use of the word “technology” implies that I consider music to be a kind of artefact that “provides means for enacting scenarios as motivation and opportunity arise” (DeNora, 2000, p. 36). DeNora applies the concept of “affordance” to describe music's certain kinds of uses or interpretations. We might also say that inherent to music are metaphorical potentialities (Bonde, 2002) that are interpreted by the given individual and then put into action.

### Music as a social and emotional resource

When discussing the possible health effects of musicking, we also have to consider psychological and more general bio-medical aspects of this act. For example, there might be links between the act of musicking and processes related to bodily functioning, such as breathing or physiological correlates to conditions of stress, anxiety, and pain (see Hanser, 2010). In what follows, I will specifically address psychological aspects of the preceding narratives, focussing in particular upon social networking and emotional regulation.

In the first example, we met a woman who had established a choir and thereby not only created a social network but also gained great pleasure in singing and learning about music. Research on the links among singing, choir participation, and health is now quite extensive (see Balsnes, 2010; Clift, Hancox, Staricoff, & Whitmore, 2008; Clift et al., 2010) and indicates a host of related benefits. Singing in choirs affects our bodies (releasing tensions, increasing breathing capacity, maintaining our muscular and skeletal systems, giving us the experience of physical well-being) and our psychological or emotional states (releasing emotions and reducing stress, enhancing happiness and positive emotions, increasing energy levels and even producing measurable therapeutic results in relation to, for example, depression). Singing in choirs also affects our cognitive functions (stimulating attention, concentration, memory and learning abilities, our experience of mastery, and our learning and development, and increasing our self-confidence) and our existential dimensions (contributing to our experience of meaning, of being absorbed, of being part of something larger, of coherence in life, of personal transcendence, and of being engaged in something meaningful or beautiful).

Concerning the social benefits of singing in a choir, we saw in the first example how this new network gave the informant support and provided new friendships. In social research, there is growing interest in the ways in which our “social capital”—how well-integrated we are in society, and the extent of our social contacts—may constitute a health resource as well. Putnam (2000) has shown that societies with decreasing communal activities and shrinking social networks soon reveal a dip in statistics concerning health. For a discussion of the relationship between social capital and health, see Procter (2004), Stige and Aarø (2012), and Helsedirektoratet (2010).

In all of the narratives, the impact of musically induced emotions was important. In recent literature on music and emotion, researchers also seem to agree that the health aspects of this interaction will receive more scholarly attention in the years to come (Juslin & Sloboda, 2010). The relationship between music and emotion is complex and entails biological explanations that draw on our knowledge of how music is processed in different parts of the brain, as well as our individual learning histories in relation to musical structures (see also Peretz, 2010, or Juslin, Liljeström, Västfjäll, & Lundquist, 2010). In our examples, we are not so concerned about what may happen with music and emotional processing on the subcortical level but rather with cortical processing as we try to process the musical meanings. However, in either case, the level of conscious processing that accompanied the music listening indicates that higher brain functions must be involved.

Other explanations for how music might elicit emotions point to the role of learning and conditioning in our attempts to apply music-generated emotions to our larger emotional regulation. In the third narrative above, the woman used the metaphor of a chest of drawers when she tried to describe the ways in which different music could render different emotions according to her needs. We saw her explore and apply music based on her associations via her chest of drawers but also discover new aspects of music's expressiveness that she applied to her special situation as well. We also saw her repeat the process in order to obtain specific benefits from it.

In cases such as these, neither neuropsychological descriptions nor individual learning histories can fully explain how music was felt to “match” emotions or inner states. Research on music and emotion points to an element of emotional contagion when listening to or playing music. The woman in the third narrative felt strongly, for example, that the music of Marianne Faithfull could express what she herself was feeling, and then the woman took over Faithfull's emotional insight and used it for herself. The woman in the second narrative, who played Chopin, could also have been exposed to a certain contagion thanks to the changing character of this music as well. In each situation, both the character or structure of the music and the particular performance could have given rise to the concomitant feelings aroused in the situation, or to a particular metaphor that could then be applied to the situation.

### A supportive self object

In some of the narratives, it became obvious that music offered itself as a mirror of an inner state and thus helped the person to recognize, identify, distinguish, express, and finally tolerate the emotion that was produced in his/her interaction with the music. Music, for example, was very helpful in the emotional work that was necessary to integrate feelings of loss with other concomitant emotions. Self psychologist Heinz Kohut held the view that the integration of affect states is central to the development of self-regulatory capacities, and to the self-experience (Monsen & Monsen, 1999; Ruud, 2010). Within the theory of affect consciousness, it is fundamental to allow people to experience and learn to tolerate their emotions fully. In their work on affect theory, Monsen and Monsen present a model of affect consciousness and how it can support the understanding of therapeutic processes. The authors describe the concept of affect consciousness as the mutual relationship between the activation of basic affects and the individual's capacity to consciously perceive, reflect on, and express these affect experiences.

Music seems to offer itself as a safe and positive “self object”, something that one can trust and even interact with that supplies the strength to work through challenges.

### Musical competency

From these narratives, we find that some sort of musical training, interest, skill, or competency promotes or intensifies the health potential of music. In the third narrative, the woman had cultivated her musical interest throughout her life and acquired a large collection of CDs along the way. Some of the other informants described musical skills that made it possible for them to express or perform their emotions in different musical contexts. We also saw that a variety of genres, artists, or musical forms could serve health functions, and that personal musical choices became crucial to these ends.

On the other hand, we know from the literature (Gabrielsson, 2008) that people with little background, competency, or even interest in music can have powerful musical experiences. It would appear that we need more narratives in order to fully comprehend the role of musical training and background in such music-related self-care.

### A special place, a special *ritual*

I would also suggest that health-related musicking generally takes place within a certain ritual structure—not, as Stige (2012, p. 189) discusses, rituals as strict formal procedures or stereotyped actions, but “individualized rituals” (ibid.) within which a person positions the body or otherwise prepares him/herself through a certain cognitive schema to enter a certain mode of experience. Building up expectations via ritual may facilitate the musicking experience and canalize the emotions. In the third narrative, for example, the woman told me that she had a certain chair or sofa she used for listening. She also described entering into a sort of hypersensitive state, where she felt like she had antennas for musical emotions. It is also true, as we indicated in the previous section, that musical experiences can come to us “from out of nowhere” as well, especially when we do not exercise any type of specific expectations while anticipating them.

### Health and locus of control

I would also propose that, in order to realize music's self-care functions, one should have a positive attitude towards the maintenance of one's health, an attitude that is rooted in a belief system encompassing the possibility of personal control over one's health situation (Ogden, 2000). In the preceding narratives, we saw the informants actively explore music in order to overcome a painful situation, deal with their grief and anxieties, improve bodily functions, reduce stress, or enhance their self-esteem and social skills.

To take grief work as an example, psychologist and music therapist Unni Johns describes some of the conditions involved in processing grief (Johns, 2011). First of all, one must achieve and maintain a safe situation. We saw in the first narrative that music allowed the woman to surrender herself without fear of judgment. Johns also notes the importance of agency and emotional regulation, both of which were prominent in the narratives, not least in the way the informants felt they could control their own situations and influence their own emotional states through musical means. Even though music could provoke unpleasant emotions as well, the woman in the third narrative, for example, was able to maintain control of it through her own choices of what to listen to, where to sit, how long to listen and so on (see Skånland, 2012, for a discussion of regulation and control through music).

Although we might again argue that these are not *necessary* conditions for individualized health musicking, they are likely constructive parts of a reflexive practice whereby individuals intentionally seek to maintain their health through a defined long-term program of musical self-care.

There seem to be two main notions of “health” in common discourse. In a biomedical context, to be in a state of health seems to mean to live without disease. On the other hand, many people seem to think of health as a state of being which implies a certain surplus of energy and contentment (Ogden, 2000, p. 43). This more positive sense of health tends to equate it with “quality of life”, another rather debatable concept with the field of medicine and health psychology (see Ogden, 2000, chapter 14). As I have argued elsewhere (Ruud, 1998; 2001), our perception of quality of life has many subjective dimensions and is subject to the values projected by various professions: medical doctors value and protect the body and life; social workers are concerned about the democratic distribution of welfare goods; psychologists are fundamentally concerned about human rights and dignity.

Whatever we may think about this question, I have always held the belief that we make music to serve some human need, and that it therefore surely has an impact upon quality of life, as a:

1. provider of vitality—that is, emotional stimulation and expression;
2. tool for developing agency and empowerment;
3. resource in building social networks; and
4. way of providing meaning and coherence in life (see Ruud, 1998).

To the extent that musicking involves addressing some of these needs, we might argue that it provides a better quality of life, and thus better health. Looking back to the six informants here, it is easy to see how their health musicking touched all of these areas.

## Conclusion

As suggested in “Introduction” section, the aim of this article is to investigate what we might learn from such stories in terms of mapping some of the conditions that seem necessary for music to promote health or serve as a cultural immunogen. As we gather more narratives about how music is used in the everyday life in order to regulate, maintain, and improve health, we will learn more about the contextual and generative factors behind this immunogen practice. The six factors isolated from this small qualitative project may suggest future directions for the development of a better understanding of how everyday musicking may become a part of how we maintain, promote, or improve our health and life quality.

## Footnotes

1 The term “musicking” was introduced by Christopher Small (1998) to define “music” as an activity, a verb, something that takes on its meaning depending on the context.
